# Notes and News

**Published:** 1902-03-22

**Authors:** 


### Notes and News.

### HOSPITAL MEETINGS.

Hospital Saturday Fund.—Annual Meeting, Saturday, March 22nd. at the Mansion House. at 4 p.m.

North-West London Hospital.—Annual Meeting, Monday, March 24th, at 5 p.m.

British Lying-in Hospital.—Annual Meeting, Monday, March 24th, at 5 p.m.

London Temperance Hospital.—Annual Meeting, Monday, March 24th, at 5 p.m.

Royal Sea-Bathing Hospital, Margate.—Annual Meeting, Monday, March 24th, at 30 Charing Cross, at 3.30 p.m.

St. Peter's Hospital for Stone.—Annual Meeting, Tuesday, March 25th, at 5 p.m.

Poplar Hospital.—Annual General Meeting, Tuesday, March 25th, at the conclusion of the Special Meeting fixed for 5 p.m.

National Hospital for Paralysed.—Annual Meeting, Wednesday, March 26th, at 3 p.m.

The committee of Our Dumb Friends' League have placed the first ambulance for horses at the disposal of the metropolis. It has been exhibited at Messrs. Tattersalls.

As a result of the opening ceremony of the David Lewis Northern Hospital at Liverpool by the Princess Louise, £1,600 was contributed in purses to the Princess.

For some time past the London County Council has been considering the equipment of an ambulance service for London, and it has been decided to establish a horse ambulance system.

The plans and estimates for a new Royal Infirmary at Manchester are to be submitted at an early date. The infirmary is to be built on the present site, and will not be on the pavilion system.

The Metropolitan Convalescent Institution proposes to build another home at Bexhill—for men only, reserving the present home for women—out of a fund which could be applied for such a purpose, bequeathed to the seaside branch of the institution by the late Mr. Henry Vaughan. An additional £2,000 a year will be required for the maintenance of the new home and the Board appeal very earnestly for increased subscriptions and donations.

In the stricken village of Schemacha in Russia, both typhus and diphtheria have broken out amongst the inhabitants.

An Anglo-American Hospital is to be built at Cairo. The King and Queen will give it their patronage. Sir Edward Cassel has promised £5,000 towards it, if the same sum is contributed from other sources.

The hospital ship *Maine* is now used as a naval collecting hospital in the Mediterranean. It conveys the sick in the squadrons to the Royal Naval Hospital at Malta, and also brings patients home to England.

The Hospital for Sick Children will hold a Coronation Bazaar in the gardens of the hospital during the first week in July. The Queen has promised to be present if possible, and the list of stall-holders and patrons is large and distinguished.

In Austria a lady has succeeded in getting substantial damages out of a railway company for injuries to her nerves caused, she asserted, through the train suddenly entering a tunnel, the carriage being unlighted. She gained her case on the plea that the carriage should have been properly lighted.

The crusade against cancer is being actively pushed in Germany. The Government is about to establish two institutes for its treatment in Berlin, whilst £7,500 annually for three years has been contributed from private sources for investigations to be carried out under the direction of Professor Ehrlich, of Frankfort on-the-Main.

Lord Ilchester presided on Tuesday at a county meeting at Dorchester to consider the advisability of joining with Devon and Cornwall in the erection of a sanatorium for consumption. It was moved by Lord Portman, and seconded by Lord Stalbridge, and agreed, that a county committee should be formed to decide whether a separate institution should be established for Dorset, or whether that county should combine with others. Lord Ilchester has offered a site for a separate sanatorium.

Mr. Rockfeller has offered £200,000 to Harvard University to complete the scheme towards which Mr. J. P. Morgan gave a like sum. Mr. Rockfeller's gift is accompanied by the stipulation that £100,000 shall be contributed by the public. Mrs. James Stillman, of New York, has already given £20,000 to endow one professorial chair, and another gift of £15,000 will be used for a like purpose. Harvard has also received about £10,000 for the investigation and treatment of chronic diseases.

In spite of the fact that London itself is little disturbed by the presence of small-pox, in a small degree, in its midst, the provinces continue to show alarm, and shun its contagion. The medical officer for Nottingham went so far as to post up a warning to intending excursionists against visiting London, with the result that a popular football match at Tottenham appreciably lessened its crowd of spectators. The number of cases in the Metropolis has shown a steady decrease during the past week.

